# Enhancing immunity against *Candida albicans* infections through TIGIT knockout

**DOI:** 10.1128/mbio.01165-24

**Published:** 2024-08-07

**Authors:** Ahmed Rishiq, Mingdong Liu, Ofer Mandelboim

**Affiliations:** 1 The Concern Foundation Laboratories at the Lautenberg Center for Immunology and Cancer Research, Institute for Medical Research Israel Canada (IMRIC), Hebrew University-Hadassah Medical School, Jerusalem, Israel; Dartmouth College, Hanover, New Hampshire, USA

**Keywords:** TIGIT knockout, *Candida albicans*, fusion protein, systemic candidiasis, agglutinin-like sequences

## Abstract

T cell immunoreceptor with immunoglobulin and immunoreceptor tyrosine-based inhibitory motif (ITIM) domain (TIGIT) is an inhibitory receptor expressed by T and natural killer cells. Here, we used TIGIT knockout (KO) mice to demonstrate that mouse TIGIT directly interacts with *Candida albicans*. Reduced fungal growth and colonization were observed when TIGIT-KO splenocytes were co-cultured with *C. albicans* compared to the wild type (WT). In a systemic candidiasis model, TIGIT-KO mice exhibited improved survival and reduced body weight loss compared to WT mice. Organ-specific fungal burden assessment revealed significantly lower fungal loads in the kidneys, spleen, and lungs of TIGIT-KO mice. Finally, we show that the agglutinin-like sequence proteins ALS6, ALS7, and ALS9 of *C. albicans* are ligands for TIGIT and that the absence of these proteins abolishes the TIGIT effect *in vivo*. Our results identify the significance of TIGIT in modulating host defense against *C. albicans* and highlight the potential therapeutic implications for *C. albicans* infections.

**IMPORTANCE:**

Our results identify the significance of T cell immunoreceptor with immunoglobulin and ITIM domain in modulating host defense against *Candida albicans* and highlight the potential therapeutic implications for *C. albicans* infections.

## INTRODUCTION

Fungal infections are becoming more prevalent, impacting millions of individuals annually. It is widely recognized that the initial defense against *Candida albicans* infection primarily relies on innate and adaptive immune cells, notably neutrophils, dendritic cells, and macrophages, serving as the frontline responders (1). For decades, the antifungal role of natural killer (NK) cells has been underestimated. However, *in vitro* studies have highlighted that NK cells affect *C. albicans* infection, both directly through cytotoxic killing and indirectly through the production of cytokines (2). Adaptive immune cells specifically involving Treg, Th1, Th2, and mainly Th17 cells were also shown to control *C. albicans* infection (3, 4). Immune inhibitory checkpoints have been identified as promising targets for inhibiting tumor progression (5). These checkpoints are often expressed on exhausted immune cells and prevent the full action of these cells, a phenomenon shared between invasive candidiasis and cancer (6). Indeed, single-cell RNA sequencing demonstrated that following stimulation with *C. albicans* in healthy individuals, a co-induction of inhibitory molecules such as PD-1, CTLA-4, and LAG-3 was observed in Th cells, cytotoxic T lymphocytes, and mononuclear phagocytes (7). Thus, checkpoint blockade could be considered as a potential strategy to enhance the immune response against fungal infections, including those caused by *C. albicans*.

T cell immunoreceptor with immunoglobulin and ITIM domain (TIGIT) is an inhibitory receptor that is expressed by innate and adaptive lymphocytes. Blocking TIGIT can boost antitumor T and NK cell responses (8). There have been reports indicating that TIGIT pathway modulation is implicated in altering the phenotype and cytokine profile of T cells during viral infections, such as influenza and lymphocytic and choriomeningitis virus (LCMV) infection (9). Other studies have also investigated the role of TIGIT in the context of various *Fusobacterium nucleatum* (10), *Toxoplasma gondii* (11), and *Echinococcus multilocularis* infections (12). We have previously revealed that TIGIT interacts with *C. albicans*, and the fungal ligands responsible for this interaction belong to the agglutinin-like sequence (Als) protein family (13).

In this study, we will explore *C. albicans* infection using a TIGIT knockout (KO) mouse model, which we generated previously (14). Our aim is to assess how the absence of TIGIT impacts systemic candidiasis. Exploring the interactions between *C. albicans* and TIGIT may offer valuable insights into the broader regulation of antifungal immune responses. This research has the potential to enhance our understanding of how the immune system manages the delicate balance between controlling *C. albicans* and the potential for excessive inflammation and tissue damage.

## MATERIALS AND METHODS

### Mice

TIGIT-KO mice were generated as previously described (14). Six-to-eight-week C57BL/OlaHsd wild-type (WT) mice were purchased from Envigo. Mice were housed and bred in a specific pathogen-free unit of the animal facility of the Hebrew University of Jerusalem.

### *Candida albicans* culture, staining, and flow cytometry

*C. albicans* SC5314 and ALS6∆/∆, ALS7∆/∆, and ALS9∆/∆ were obtained as previously described (13). In brief, fungi were cultured on Sabouraud dextrose agar (SDA) (Sigma-Aldrich) plates on a stationary 30°C incubator. For yeast culturing, a toothpick was taken from colony streak and inoculated in Sabouraud dextrose broth in an overnight aerobic shaking 30°C incubator. On the next day, a 1:50 dilution was cultivated for 2–4 h. Yeast cells (0.5 × 10^6^) were counted using the Neubauer hemocytometer chamber then stained with 0.1 mg/mL of fluorescein isothiocyanate (FITC) at room temperature for 30 min then washed twice with PBS 1× and centrifuged for 4,000 rpm for 10 min. FITC-stained *Candida* was incubated with 2.5 µg of mouse TIGIT fusion protein (mTIGIT-Ig) that we have generated previously (15) on ice for 1 h, centrifuged for 4,000 rpm for 10 min, and washed with ice-cold PBS 1× twice; then, binding was detected by a secondary antibody (allophycocyanin anti-human IgG, Jackson), at a dilution of 1:200, after incubation for 30 min on ice. The following antibodies were used for staining: PE anti-mouse NKp46 (NCR1), APC anti-mouse CD4, APC anti-mouse CD8, APC anti-mouse TIGIT (Vstm3), PE anti-mouse CD4, APC anti-mouse CD3, and PE anti-mouse CD8 all from BioLegend.

### Splenocyte preparation

The mouse was euthanized by CO_2_ inhalation, placed on its back on a dissection mat, and sprayed with 70% ethanol. An incision in the abdominal area was made until the neck with a pair of straight scissors. The spleen was collected from the left side and promptly placed into Hank’s balanced salt solution (HBSS; devoid of calcium and magnesium) and supplemented with 1% heat-inactivated fetal bovine serum (FBS). Splenocytes were flushed out of the spleen by injecting HBSS with a 20-mL syringe and 26 G needle into a 50-mL tube. Cells were centrifuged, and red blood cells (RBCs) were lysed using Ack lysis buffer (0.1 5M NH_4_Cl, 0.01 M KHCO_3_, and 0.0001 M EDTA) for a duration of 5 min. Afterward, they were washed with PBS 1× and centrifuged at 300 × *g* for 5 min. The cells were subsequently placed in an IL-2 culture medium (400 IU) and allowed to incubate for 48 h before being co-cultured with *C. albicans* (13, 14).

### Co-culturing of *C. albicans* with mouse splenocytes

Splenocytes isolated from WT and TIGIT-KO were co-cultured with *C. albicans* (100:1) in U-bottomed 96-well plates for 24 h. The plate was centrifuged at 4,000 rpm for 10 min; then, distilled water was added to eliminate the splenocytes followed by serial dilution. The resulting diluted samples were then plated onto SDA (Sigma-Aldrich) plates and incubated for 24–48 h on a stationary 30°C incubator.

### Reduced viability assay

XTT [2,3-bis-(2-methoxy-4-nitro-5-sulfophenyl)-2*H*-tetrazolium-5-carboxanilide] (Invitrogen, X6493) was used to evaluate the viability of the fungal cells. Briefly, fungal cells were washed three times with 1× PBS; then, distilled water was used to dissolve the splenocytes. Subsequently, 100 µL (1 µM) of XTT/menadione was added per well then incubated for 1–2 h at 37°C in the dark. The plate then was centrifuged, 80 µL was transferred from each stained well to a new plate, and data were measured using the plate reader (Spark multimode microplate reader) at a wavelength of 490 nm. The percentage of reduced viability was determined using the following equation: reduced viability percentage = [(*Candida* only − splenocytes: *Candida*)/(*Candida* only − splenocytes only)] × 100.

### Systemic candidiasis model

To induce systemic candidiasis, a total of 0.5 × 10^6^ *C. albicans* SC5314, ALS6∆/∆, ALS7∆/∆, and ALS9∆/∆ cells were intravenously (IV) injected into both WT and TIGIT-KO mice. The mice were restrained using a mouse vein restrainer for tail vein injections. Subsequently, conidia were injected into either the left or right lateral caudal vein. Body weights as well as the survival rate were subsequently recorded on days 0 and 1, 4, and 5 post-infection. To investigate the organ fungal burden, a lethal dose (0.65–0.7 × 10^6^) of *C. albicans* was IV injected for 48 h, and then, organs were harvested, processed, and passed through a 70-µm cell restrainer in 1× PBS. Afterward, the organ solution was serially diluted and plated on SDA plates, where it was allowed to incubate for 24–48 h before fungal colonies were counted.

To isolate lymphocytes from healthy and *C. albicans*-infected mouse organs, mice were infected; then, splenocytes were isolated by digesting the whole spleen placed in a 70-µm restrainer with FACS buffer (2 mM EDTA, 0.5% bovine serum albumin [BSA] in PBS 1×). Cells were centrifuged at 1,600 rpm for 5 min then counted and stained. Blood lymphocytes were harvested from the heart blood via cardiac puncture. RBCs were then lysed using ACK lysis buffer and centrifuged at 1,600 rpm/5 min. Lungs were finely minced and digested at 37°C in a digestion solution containing 0.5 mg/mL of collagenase V (Worthington LS004188) and 0.1 mg/mL of grade II DNAse I (Sigma-10104159001) for 30–45 min with intermittent shaking. The digested pieces flowthrough passed through a 70-µm cell restrainer and then centrifuged at 1,600 rpm for 5 min. Cells were then resuspended in the desired volume of the FACS buffer (200–600 µL). Kidneys were cut into small pieces and were added to 5 mL of digestion solution (20 mM HEPES, 0.3 mg/mL Liberase TL [Roche, 05401020001], and 0.1 mg/mL of DNase I in RPMI-FBS free medium) at 37 °C for 30 min with intermittent shaking. An equal volume with RPMI + 10%fetal calf serum (FCS) was added, and supernatants were placed on a 70-µm cell strainer and centrifuged at 1,600 rpm for 5 min. The pellet was resuspended in 40% Percoll (Sigma-Aldrich, Cytiva 17-0891-01) prepared in RPMI serum-free medium, placed on 3 mL of 70% Percoll in 1× PBS, and then centrifuged at 800 rcf for 30 min without break. Liver lymphocytes were prepared as described previously (16)

### Statistics

Statistical analysis and graphs were conducted using GraphPad Prism Software version 9 (GraphPad Software). Statistical significance and differences were determined using unpaired Student’s *t*-test and the Mann–Whitney test. To assess the significance of the survival assay, a Mantel–Cox log-rank test was employed. For multiple comparisons, two-way ANOVA followed by Sidak’s multiple comparisons post-test was used. *P*-value was considered significant at *P* < 0.05.

## RESULTS

### Murine TIGIT knockout (TIGIT-KO) has impact on both viability and colonization of *C. albicans*

**Fig 1:**
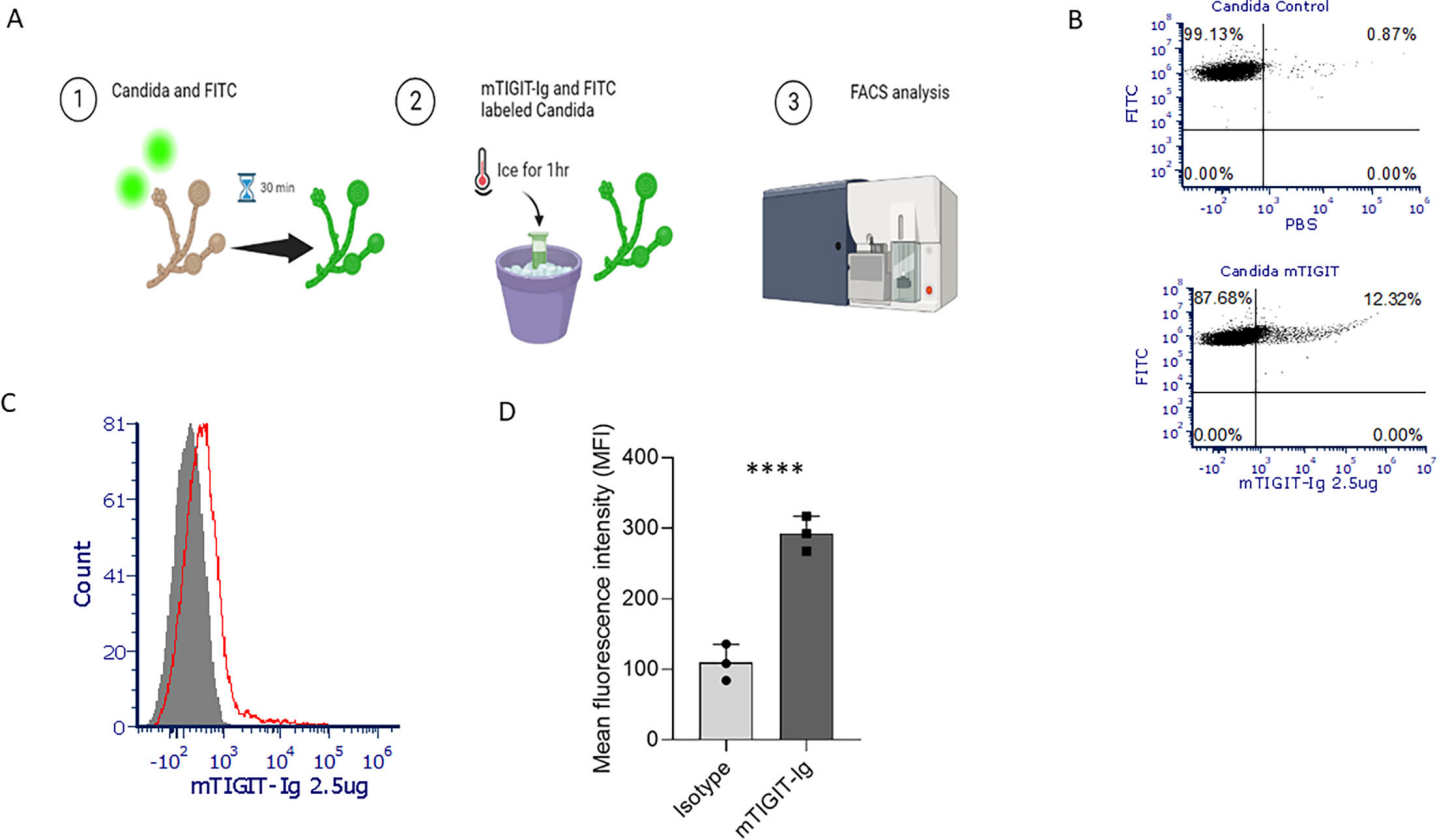
Murine TIGIT (mTIGIT) binds *C. albicans*. (A) Schematic view of FITC-labeled *C. albicans* (1), incubated with mTIGIT-Ig for 1 h on ice (2), and then analyzed by FACS (3) (created with BioRender.com). (B) Representative dot plot of analyzed FACS without (upper) and with (lower) mTIGIT-Ig. (C) FACS histogram of background (gray filled) and mTIGIT-stained *C. albicans* (red histogram). (D) FACS analysis quantification of control vs mTIGIT-Ig-stained *C. albicans*. The presented values represent the mean ± SD obtained from three independent experiments. ****P < 0.0001.

**Fig 2:**
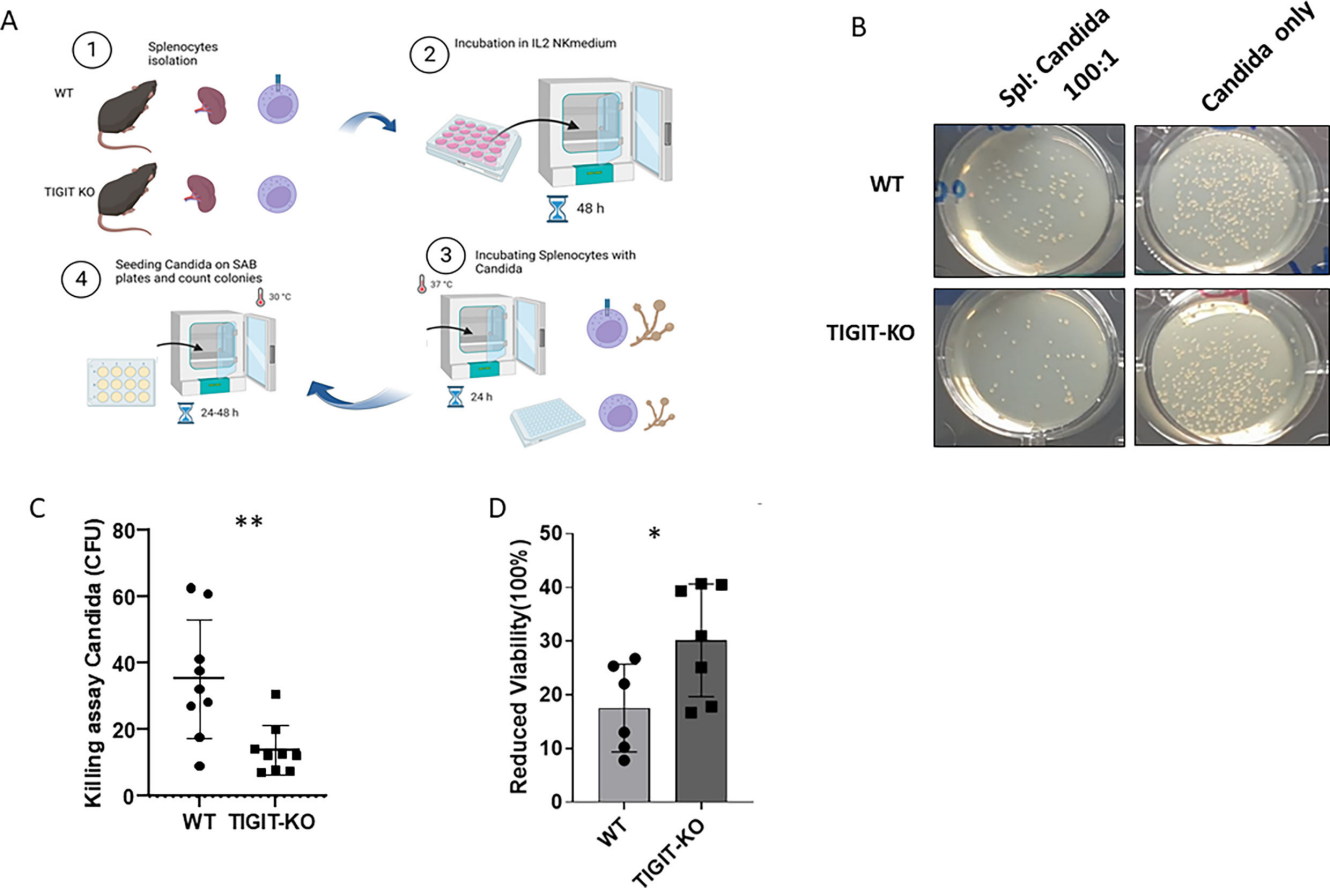
Knockout of TIGIT resulted in reduced *C. albicans* growth. (**A**) Schematic representation depicting the experimental procedures for the co-culture of mouse splenocytes with *C. albicans*. Splenocytes were extracted from both WT and TIGIT-KO mice (1). They were then incubated in a medium containing IL-2 for 48 h (2). Following this, *C. albicans* was incubated with the splenocytes for 24 h at 37°C (3). The co-cultured splenocytes and *C. albicans* were subsequently plated on SDA plates and left to grow at 30°C in a stationary incubator for 24–48 h (4) (created with BioRender.com). (B) A representative image displays the reduced colonies formed 24–48 h after the incubation of co-cultured *C. albicans* with both WT and TIGIT-KO splenocytes on SDA (Spl: *Candida* 100:1) as described in panel **A** compared to the cultured *C. albicans* without splenocytes (*Candida* only) (left panel). (**C**) Quantification of the number of the CFU of the grown *C. albicans* following 24–48 h. (D) Reduced viability percentages of *C. albicans* incubated with splenocytes isolated from WT and TIGIT-KO mice determined using XTT assay. The same procedures were conducted as in panel A, but instead of plating co-cultured *C. albicans* and splenocytes, XTT/menadione was added and then incubated for 1–2 h at 37°C. Percentage was calculated as described in Materials and Methods. *P < 0.05 and **P ≤ 0.01. We performed the experiment three times using different mice.

To test whether mouse TIGIT can directly interact with *C. albicans*, fungal cells were first labeled with FITC for better visualization in FACS. Subsequently, these stained fungal cells were incubated with mouse TIGIT fusion protein (mTIGIT-Ig) for 1 h on ice and then analyzed by FACS (Fig. 1A). As can be seen, mTIGIT interacts with *C. albicans* (Fig. 1B [dot plot], Fig. 1C [FACS histograms], and Fig. 1D [quantification]). To determine the functional consequences of TIGIT interaction with *C. albicans,* we used the TIGIT-KO mice generated by us previously (14). Splenocytes were collected from both WT and TIGIT-KO mice. These splenocytes were incubated with IL-2 for 48 h and then incubated with *C. albicans* for 24 h, followed by plating onto SDA for subsequent 24–48 h (Fig. 2A). The assessment of cytotoxicity was conducted by enumerating the colony-forming units (CFU) that developed on SDA (Fig. 2B). As can be seen, reduced growth of *C. albicans* was observed when the *C. albicans* were co-cultured with splenocytes from TIGIT-KO mice as compared to those isolated from WT mice (Fig. 2C).

To confirm our results, fungal cell colonization was further evaluated using the previously described procedures in Materials and Methods but employing a different method. However, in this case, instead of plating *C. albicans*, we performed an XTT viability assay. Reduced viable fungal cells were observed in the TIGIT-KO splenocytes compared to the WT group (Fig. 2D).

### TIGIT-KO reduced *C. albicans* pathogenicity and enhanced survival *in vivo*

**Fig 3:**
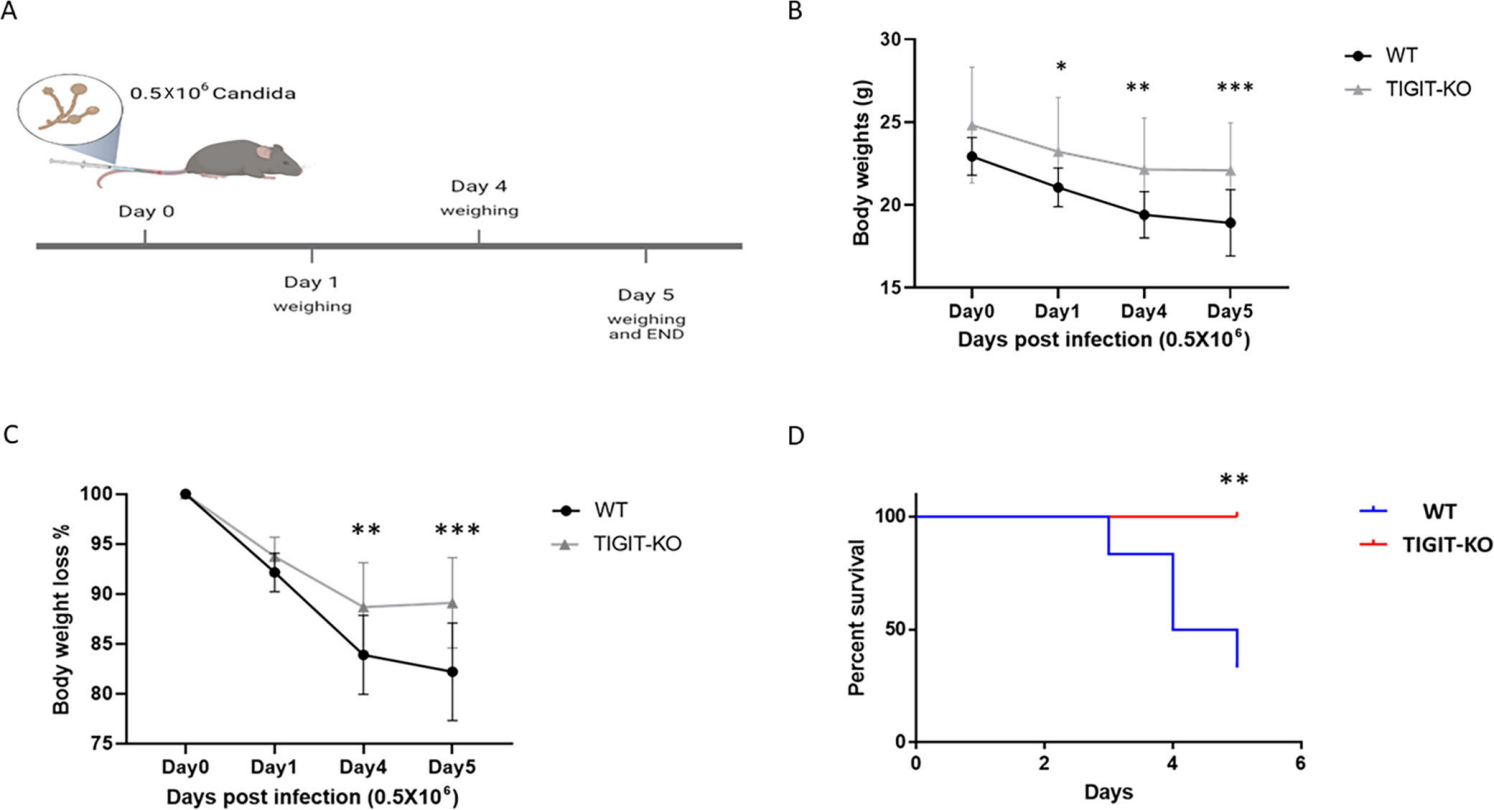
TIGIT knockout reduced invasive candidiasis. (**A)** An experimental design diagram depicts the mouse model of systemic candidiasis (created with BioRender.com). The mice were weighed at day 0 and then IV injected with 0.5 × 10^6^ *C. albicans* at day 0. Subsequently, the mice’s weights were monitored up to day 5, after which they were euthanized. (B) Body weight in grams of both WT and TIGIT-KO mice recorded on days 0, 1, 4, and 5 following the injection of *C. albicans*. (C) Percentages of body weight loss of WT and TIGIT-KO. Day 0 was considered as 100%. (D) Kaplan Meier survival analysis graph of WT (blue line) and TIGIT-KO (red line) mice infected intravenously with *C. albicans* for 5 days. The mean value ±SD of three independent experiments is presented. *P < 0.05, **P ≤ 0.01, and ***P ≤ 0.001. We performed the experiment three times using different mice.

We next sought to investigate the influence of TIGIT absence on systemic candidiasis. To do so, *C. albicans* was intravenously inoculated into WT and TIGIT-KO mice, and body weight and survival were monitored daily for 5 days following infection (Fig. 3A). As shown in Fig. 3B and C, the WT mice group showed a decreased overall body weight, along with an increased percentage of body weight loss. This phenomenon became noticeable on the first day following the fungal infection and progressively worsened until day 5. However, TIGIT-KO mice showed enhanced body weight on day 4 and exhibited fewer symptoms over time as compared to the WT group. Subsequently, the survival of both WT and TIGIT-KO mice was assessed and compared. Importantly, TIGIT-KO had a better survival rate compared to the WT mice, and all TIGIT-KO mice survived the infection (Fig. 3D).

**Fig 4:**
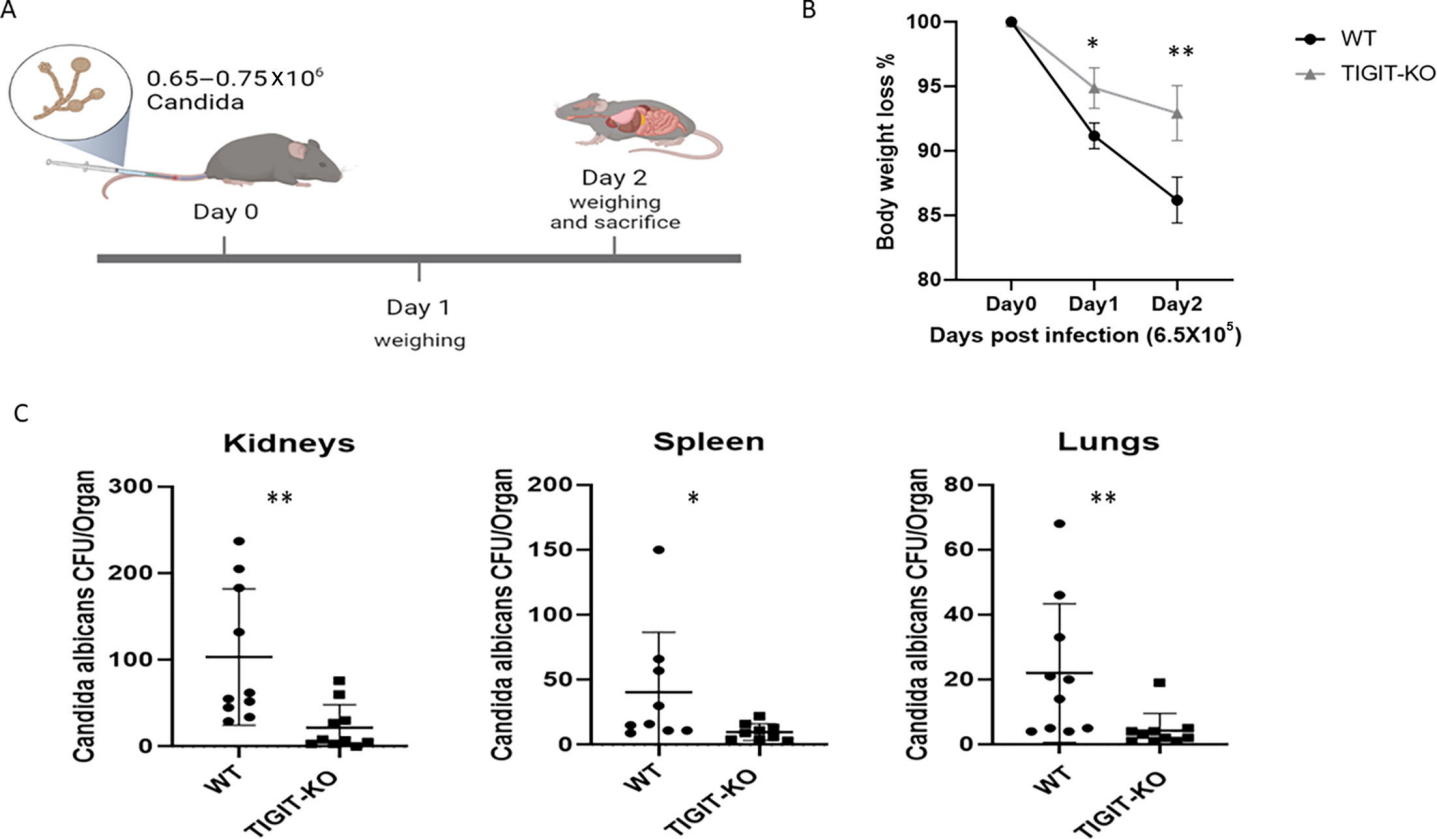
Reduced *C. albicans* burden in the organs of TIGIT-KO mice. (**A**) Both WT and TIGIT-KO mice received a high dose of *C. albicans* injection (ranging from 0.65 to 0.75 × 10^6^), and mice were sacrificed on day 2 to assess the fungal burden in mouse organs (created with BioRender.com). (B) The percentage of body weight loss was monitored in both WT and TIGIT-KO mice following *C. albicans* infection. (C) *C. albicans* burden in kidneys, spleen, and lungs 48 h post-IV infection of WT and TIGIT-KO mice. Organs were harvested, processed, and then seeded on SDA for 24–48 h. Results are shown as the mean value ±SD (*n* = 9–10); mice were examined from three independent experiments. *P < 0.05 and **P ≤ 0.01.

The fungal burden within the organs was next evaluated. Mice received a lethal dose of *C. albicans* and were sacrificed on day 2, as illustrated in Fig. 4A. Forty-eight hours following *C. albicans* infection*,* there was a noticeable reduction in body weight in WT mice compared to TIGIT-KO mice (Fig. 4B). We next harvested and processed the kidneys, spleen, and lungs of the infected mice, and these were seeded on SDA plates and incubated for 24–48 h. WT mice exhibited a significantly higher fungal burden in contrast to TIGIT-KO mice in all organs tested (Fig. 4C). Taken together, these findings demonstrate that TIGIT knockout enhances the elimination of *C. albicans* and reduces the overall fungal burden.

To examine TIGIT expression and evaluate the percentages of tissue-resident lymphocytes, we extracted cells from various organs of both uninfected, healthy mice and those infected with *C. albicans*, encompassing both WT and TIGIT-KO mice. In splenocytes, an elevated percentage of T helper CD4+, T cytotoxic CD8+, and NK cells was noted in both WT and TIGIT-KO mice following infection. However, no difference was observed between the control and infected groups in both WT and TIGIT-KO mice (Fig. S1A through C, left panel). In the WT mouse group infected with *C. albicans*, an upregulation of TIGIT expression was observed on CD4+, CD8+ T, and NK cells compared to the uninfected group (Fig. S1A through C, middle panels). Mean fluorescence intensity (MFI) was increased in CD4+ and NK cells (Fig. S1A and C, middle panels). However, this increase was not observed in CD8+ TIGIT-expressing cells (Fig. S1B, right panel). Among blood lymphocytes, only the percentages of NK cells were increased in both WT and TIGIT-KO mice following infection with *C. albicans* (Fig. S2C, left panel). Furthermore, the percentage was also elevated on NK cells in the infected WT mouse group compared to the uninfected group (Fig. S2C, middle and right panels). The percentages of CD4+ and CD8+ T cells (Fig. S2A and B, left panels) as well as TIGIT expression (Fig. S2A and B, middle and right panels) were similar. No change was observed in the CD4+, CD8+, and NK cell percentages among lymphocytes isolated from the lungs in both infected WT and TIGIT-KO mice (Fig. S3A through C, left panels). Nevertheless, there was a significant increase in TIGIT expression in CD4+ and CD8+ T cells, as well as in the percentages and MFIs of NK cells (Fig. S3A through C, middle and right panels). No change in CD4+ and CD8+ T cell percentages was observed after the infection of both WT and TIGIT-KO mice (Fig. S4A and B left panels) while NK cell percentages isolated from kidneys of infected WT and TIGIT-KO groups were elevated (Fig. S4C, left panel). Interestingly, there was an increase in the percentages of TIGIT expression in CD4+, CD8+, and NK cells (Fig. S4A through C, middle panels); this increase was not reflected in the MFI values for these cell types (Fig. S4A through C, right panels). Liver CD4+ T cells showed increased percentages in both WT and TIGIT-KO infected mice (Fig. S5A, left panel). There was a significantly reduced percentage of CD4+ T cells-expressing TIGIT following infection (Fig. S5A, middle panel). Conversely, opposite results were observed in MFI values (Fig. S5A, right panel). Remarkably, a higher percentage of CD8+ T cells was noted in TIGIT-KO mice compared to their WT control group (Fig. S5B, left panel), whereas reduced CD8 cytotoxic T cell and NK cell percentages were observed in both WT and TIGIT-KO mice following infection with *C. albicans* (Fig. S5B and C, left panels). In contrast, no change was observed in the percentages of CD8+TIGIT+ T cells or their related MFIs (Fig. S5B, middle and right panels), while upregulation of the TIGIT expression and MFI was observed in NK cells in response to *C. albicans* infection (Fig. S5C, middle and right panels). Collectively, these findings suggest an association between tissue-resident immune cells, TIGIT expression percentages, and systemic infection, with the absence of TIGIT-KO influencing disseminated candidiasis.

### Als protein family of *C. albicans* are mTIGIT ligands

**Fig 5:**
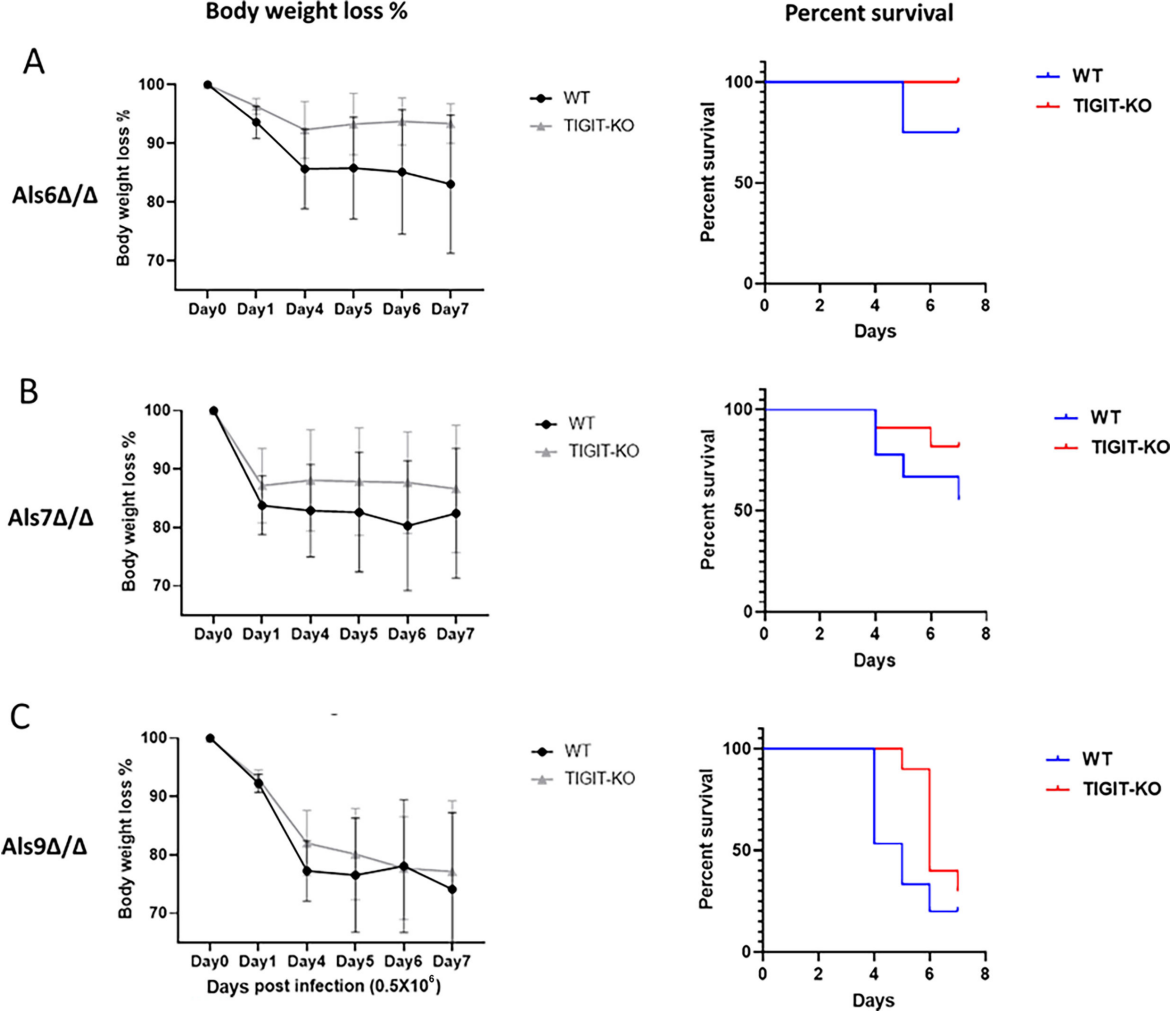
Fungal cells with deletions in ALS6∆/∆, ALS7∆/∆, or ALS9∆/∆ exhibit comparable levels of pathogenicity in both wild-type and TIGIT-KO mice. The assessment of body weight loss (left panels) and percent survival (right panels) was conducted after intravenous injection of *C. albicans* mutants with deletions in ALS6∆/∆ (**A**), ALS7∆/∆ (**B**), and ALS9∆/∆ (**C**) into both WT and TIGIT-KO mice over a 7-day period. These results are representative of two independent experiments.

We previously demonstrated that specific wall-bound proteins essential for *C. albicans* adhesion and pathogenicity, known as agglutinin-like sequences (Als), particularly ALS6, 7, and 9, act as ligands and directly interact with TIGIT (13). The role of ALS6, 7, and 9 was demonstrated *in vivo* using blocking anti-TIGIT antibodies (13). To reconfirm these results, we intravenously infected both WT and TIGIT-KO mice with *C. albicans* mutants for these proteins (ALS6∆/∆, ALS7∆/∆, and ALS9∆/∆). The weight loss and survival of the mice were monitored for 7 days following the infection. As shown in Fig. 5A through C (left panels), no notable difference in the percentage of body weight loss was observed between the WT and TIGIT-KO infected with the ALS6∆/∆, ALS7∆/∆, and ALS9∆/∆ mutants. Mouse survival was also monitored. Interestingly, infection of the WT mice with ALS6∆/∆ and ALS7∆/∆ was less severe as compared to infection with the WT *C. albicans*, whereas infection with ALS9∆/∆ was as severe (Fig. 5, right panels, and Fig. 3D). No difference in the survival of the WT and TIGIT-KO was noticed following infection with ALS6∆/∆ and ALS7∆/∆, and slightly better survival was observed when the TIGIT-KO mice were infected with ALS9∆/∆ as compared to the WT mice (Fig. 5A through C, right panels). These results indicate that these proteins are fungal TIGIT ligands and act as an *in vivo* immune-mediated evasion mechanism.

## DISCUSSION

Accumulating evidence based on previous studies supports the notion of direct interaction between *C. albicans* and CD8 as well as NK1.1+ lymphocytes (17, 18). In particular, some interactions are mediated through the inhibitory immune checkpoint like TIGIT, which is expressed by NK and T cells (13), or through co-induction of other immune receptors like PD-1, LAG-3, and TIM-3 as shown in patients suffering from invasive *C. albicans* infection (19). Upregulation of the immune checkpoints is closely associated with immune dysfunction and exhaustion, and blocking of these checkpoints is suggested as a potential treatment option (19).

In our study, we primarily showed murine TIGIT binding to FITC-labeled *C. albicans* by using a previously generated mTIGIT fusion protein (mTIGIT-Ig) (15). We found that activated splenocytes isolated from TIGIT-KO mice exhibited higher killing of *C. albicans* compared to those isolated from WT mice. Our findings revealed that *in vivo* infection of *C. albicans* in TIGIT-KO mice showed more survival and less body weight loss compared with the WT group. Moreover, the fungal burden in organs isolated from TIGIT-KO mice was also lower compared to that in organs from WT mice. This was confirmed through the culture of organ extracts and comparing the amount of *C. albicans* that grew in these cultures. Our data suggest a prominent role of TIGIT absence in enhancing fungal removal.

Generally, *C. albicans* is recognized by the innate immune cell through pattern recognition receptors such as NOD-like receptors, Toll-like receptors, and C-type lectin receptors (CLRs) (20). One such CLR, Dectin-1, which functions as an NK receptor, was shown to be involved in an immune response to *Candida* species through beta-glucans and producing TNF-α (21). Epithelial cells can also play a prominent role as an innate immune response in recognizing *Candida* through Eph2A receptor, particularly at mucosal surfaces. However, the process of epithelial activation depends on fungal morphology and candidalysin (hypha toxin) secretion (22). On the other hand, the adaptive immune response to *C. albicans* is by recognizing the antigens presented by antigen-presenting cells while B cells generate antibodies against *C. albicans* antigens (3, 20). Previously, it was found that patients lacking T cells were susceptible to candidiasis (23). Mice with both T cell and NK cell deficiencies displayed a notably high susceptibility to *C. albicans* mucosal infection. In contrast, mice with T cell deficiency alone did not exhibit the same level of susceptibility (24). Hence, the direct interaction between T and NK cells with *C. albicans* can be manipulated, potentially influencing pathogenicity.

Previously, we established that ALS6, ALS7, and ALS9 are *C. albicans* receptors and TIGIT ligands (13). Although ALS6, ALS7, and ALS9 are all involved in biofilm formation and adhesion to human epithelial cells (25, 26), clinical samples of vaginal candidiasis showed that ALS6 and ALS7 are frequently expressed less than ALS9 (27). We confirmed these findings by infecting both WT and TIGIT-KO mice with the mutant forms of ALS6, ALS7, and ALS9 (ALS6∆/∆, ALS7∆/∆, and ALS9∆/∆). Interestingly, we observed no differences in terms of body weight loss or survival percentage in the case of ALS6∆/∆ and ALS7∆/∆, but in mice infected with ALS9∆/∆, there was a non-significant trend toward increased severity. This result was aligned with our previous work where we noted that WT and TIGIT-KO mice infected with ALS9∆/∆ *C. albicans* demonstrated pathogenicity comparable to that of the unaltered ALS9 *C. albicans* strain. We also found that ALS9 binds TIGIT using microscale thermophoresis, and this result was further verified by the BW assay. Currently, we do not know why increased severity is observed following the ALS9∆/∆ *C. albicans* strain infection, and this requires investigation. Our findings suggest that these receptors play a pivotal role in *C. albicans* pathogenicity by interacting with TIGIT, potentially leading to the exhaustion of NK and T cells.

We examined the impact of infection with the wild-type strain of *C. albicans* on TIGIT expression in different organs and noted an increase in expression after the infection except for the percentages of CD4+ and CD8+ cells in circulating lymphocytes, which remained unchanged. These data suggest that NK cells, which significantly increased, remained active in fungal detection, while the adaptive immune response might require more time to be activated. Conversely, the percentage of TIGIT expression in CD4+ cells in the liver of WT mice was decreased for an unknown reason following infection with *C. albicans*, though it either increased or remained unchanged in NK and CD8+ cells, respectively (Fig. S5B, middle panel). Interestingly, we observed significantly higher percentages of CD8+ lymphocytes in the liver of the TIGIT-KO mice compared to healthy WT mice (Fig. S5B, left panel). However, despite this difference, fungal infection caused a marked decrease in CD8+ lymphocytes in both groups without any notable difference between them. It was also observed that immune cells in the lungs and kidneys (Fig. S3 and S4, respectively) of the infected mice showed no difference in their percentages. Only NK cell percentages significantly increased in both WT and TIGIT-KO infected groups without any noted difference between them.

We did not examine TIGIT expression following infection with ALS6∆/∆, ALS7∆/∆, and ALS9∆/∆ mutants. Following *in vivo* infection with these mutants, we observed they manifest different kinetics probably because these proteins play other roles in the fungi life cycle and the lower pathogenicity exerted by these strains compared to the WT *C. albicans* (Fig. 5).

An increased susceptibility to cancer was observed in *C. albicans* infection, for instance, in oral, gastric, and colorectal cancers (28). Several mechanisms have been elucidated to explain how *C. albicans* infection may contribute to cancer development. These mechanisms include fungal candidalysin toxin, carcinogenic byproducts, induction of inflammation, and through Th17 response (29, 30). In cancer patients infected with *C. albicans*, antifungal therapy is commonly employed, with fluconazole being the most frequently used antifungal agent. In cases of resistance, amphotericin B and echinocandins are alternative options (31). Our finding that *C. albicans* interacts with TIGIT has the potential to offer the benefit of using monoclonal antibodies, such as anti-TIGIT antibodies, which could serve as a remedy for cancer and may confer protection against fungal infections.

Our *in vivo* results underscore the importance of utilizing TIGIT inhibitors to enhance patient health. Given that *C. albicans* is an opportunistic pathogen and has been implicated in inflammatory bowel diseases by altering the gut microbiota (dysbiosis) and in HIV-infected patients (32), this approach holds significant promise for therapeutic interventions. Further research is warranted to comprehensively elucidate the mechanisms involved and assess the clinical implications for diagnosis and treatment.

In summary, we demonstrate that mTIGIT interacts with *C. albicans in vitro*. Furthermore, we elucidated how TIGIT absence could enhance immunity following fungal infection of TIGIT-KO in comparison to WT mice *in vivo*. Nevertheless, it is crucial to investigate the consequences of *C. albicans* interaction with the tissue-resident immune cells in organs such as the kidneys. Immunopathological damage to the kidneys in mice serves as a hallmark of fungal infection, which would finally lead to disseminated candidiasis and renal failure.

## SUPPLEMENTAL MATERIAL

Supplemental material mbio.01165-24-s0001.docx Figures S1 to S5.

ASM does not own the copyrights to Supplemental Material that may be linked to, or accessed through, an article. The authors have granted ASM a non-exclusive, world-wide license to publish the Supplemental Material files. Please contact the corresponding author directly for reuse.
