# Supplementary material for: Enhancing immunity against Candida albicans infections through TIGIT knockout
Source: mBio. 2024 Aug 7;15(9):e01165-24. doi: 10.1128/mbio.01165-24 (PMC11389390; doi:10.1128/mbio.01165-24)
Supplement: Supplemental material — Figures S1 to S5. [file mbio.01165-24-s0001.docx]

Supplementary Material

Enhancing Immunity against Candida Albicans Infections Through TIGIT Knockout

Ahmed Rishiq^1^, Mingdong Liu^1^, Ofer Mandelboim^1*^

1 The Concern Foundation Laboratories at the Lautenberg Center for Immunology and Cancer Research, Institute for Medical Research Israel Canada (IMRIC), Hebrew University-Hadassah Medical School, Jerusalem, Israel.

*** Correspondence: Ofer Mandelboim, email: oferm@ekmd.huji.ac.il**


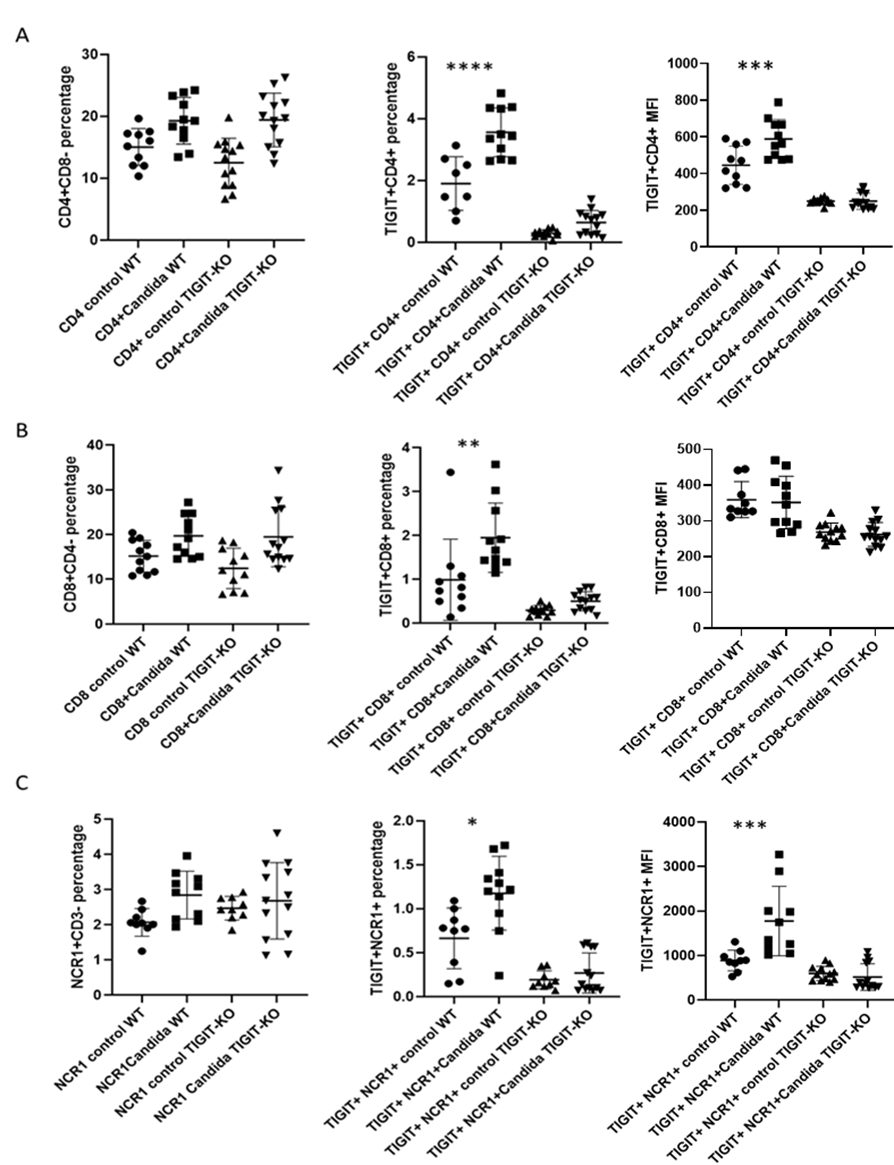


**Fig. S1.** Flow cytometry results analysis of the percentages of CD4, CD8, and NK cells, as well as TIGIT expression, were obtained for splenocytes in both healthy and *C. albicans*-infected WT and TIGIT-KO mice. A. Displays the CD4+CD8- percentage in both WT and TIGIT-KO mice (left panel), CD4+TIGIT+ percentage (middle panel) and CD4+TIGIT+ MFI (right panel) before or after *C. albicans* infection. B. Illustrates CD8+CD4- percentage in both WT and TIGIT-KO mice (left panel), CD8+TIGIT+ percentage (middle panel) and CD8+TIGIT+ MFI. And C. Shows NCR-1+CD3- percentage in both WT and TIGIT-KO mice (left panel), CD4+TIGIT+ percentage (middle panel) and CD4+TIGIT+ MFI (right panel).  *n* = 9–14 animals examined over 3 independent experiments. *P < 0.05, and **P ≤ 0.01, and *** P ≤ 0.001.


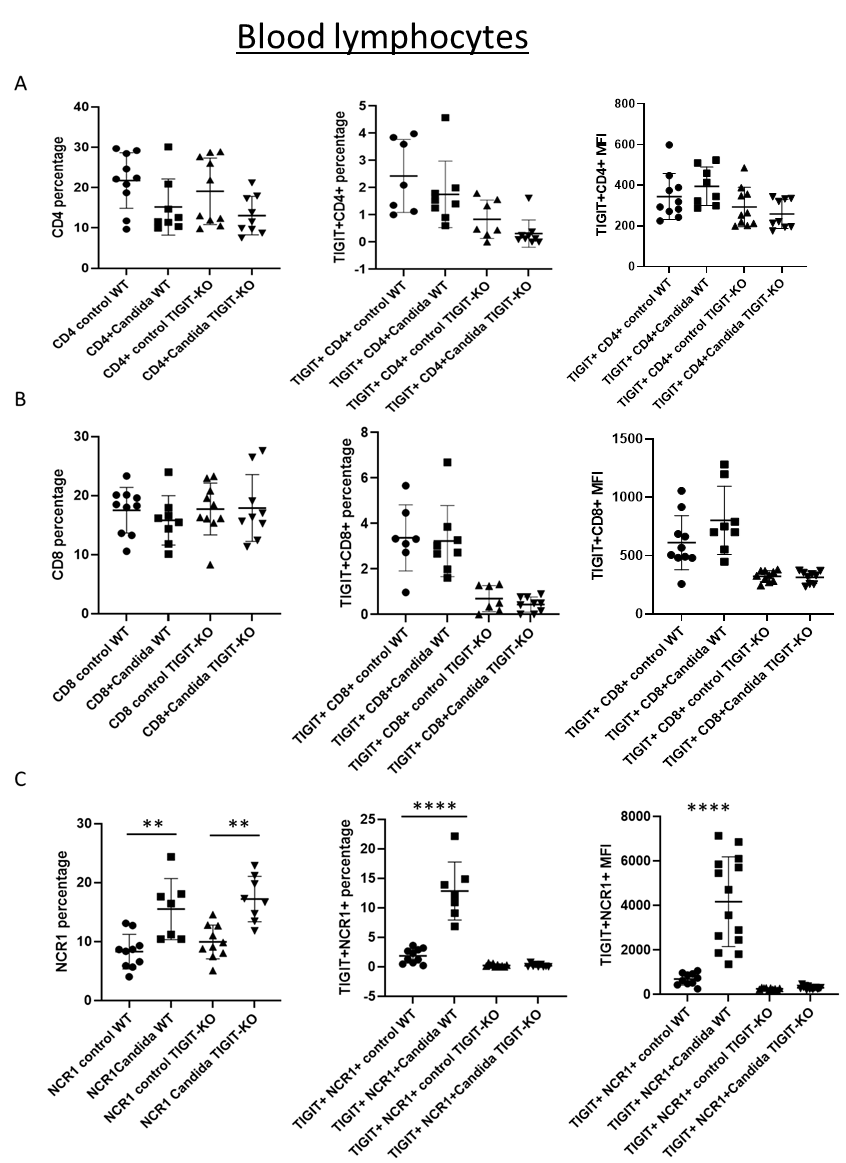


**Fig. S2.** Flow cytometry results analysis of the percentages of CD4, CD8, and NK cells, as well as TIGIT expression, were obtained for circulating lymphocytes in both healthy and *C. albicans*-infected WT and TIGIT-KO mice. A. Displays the CD4+CD8- percentage in both WT and TIGIT-KO mice (left panel), CD4+TIGIT+ percentage (middle panel) and CD4+TIGIT+ MFI (right panel) before or after *C. albicans* infection. B. Illustrates CD8+CD4- percentage in both WT and TIGIT-KO mice (left panel), CD8+TIGIT+ percentage (middle panel) and CD8+TIGIT+ MFI. And C. Shows NCR-1+CD3- percentage in both WT and TIGIT-KO mice (left panel), CD4+TIGIT+ percentage (middle panel) and CD4+TIGIT+ MFI (right panel).  *n* = 7–10 animals examined over 4 independent experiments. *P < 0.05, and **P ≤ 0.01, and *** P ≤ 0.001.


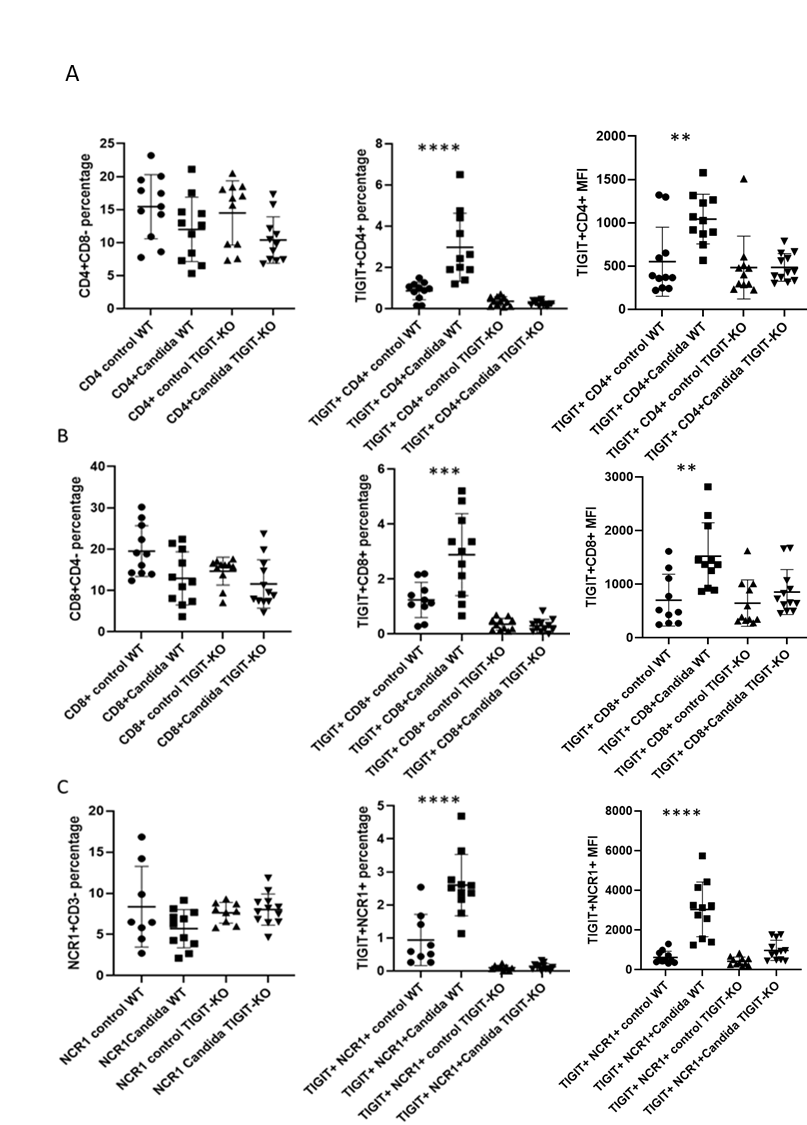


**Fig. S3.** Flow cytometry results analysis of the percentages of CD4, CD8, and NK cells, as well as TIGIT expression, were obtained for lungs in both healthy and *C. albicans*-infected WT and TIGIT-KO mice. A. Displays the CD4+CD8- percentage in both WT and TIGIT-KO mice (left panel), CD4+TIGIT+ percentage (middle panel) and CD4+TIGIT+ MFI (right panel) before or after *C. albicans* infection. B. Illustrates CD8+CD4- percentage in both WT and TIGIT-KO mice (left panel), CD8+TIGIT+ percentage (middle panel) and CD8+TIGIT+ MFI. And C. Shows NCR-1+CD3- percentage in both WT and TIGIT-KO mice (left panel), CD4+TIGIT+ percentage (middle panel) and CD4+TIGIT+ MFI (right panel).  .  *n* = 8–12 animals examined over 3 independent experiments. *P < 0.05, and **P ≤ 0.01, and *** P ≤ 0.001.


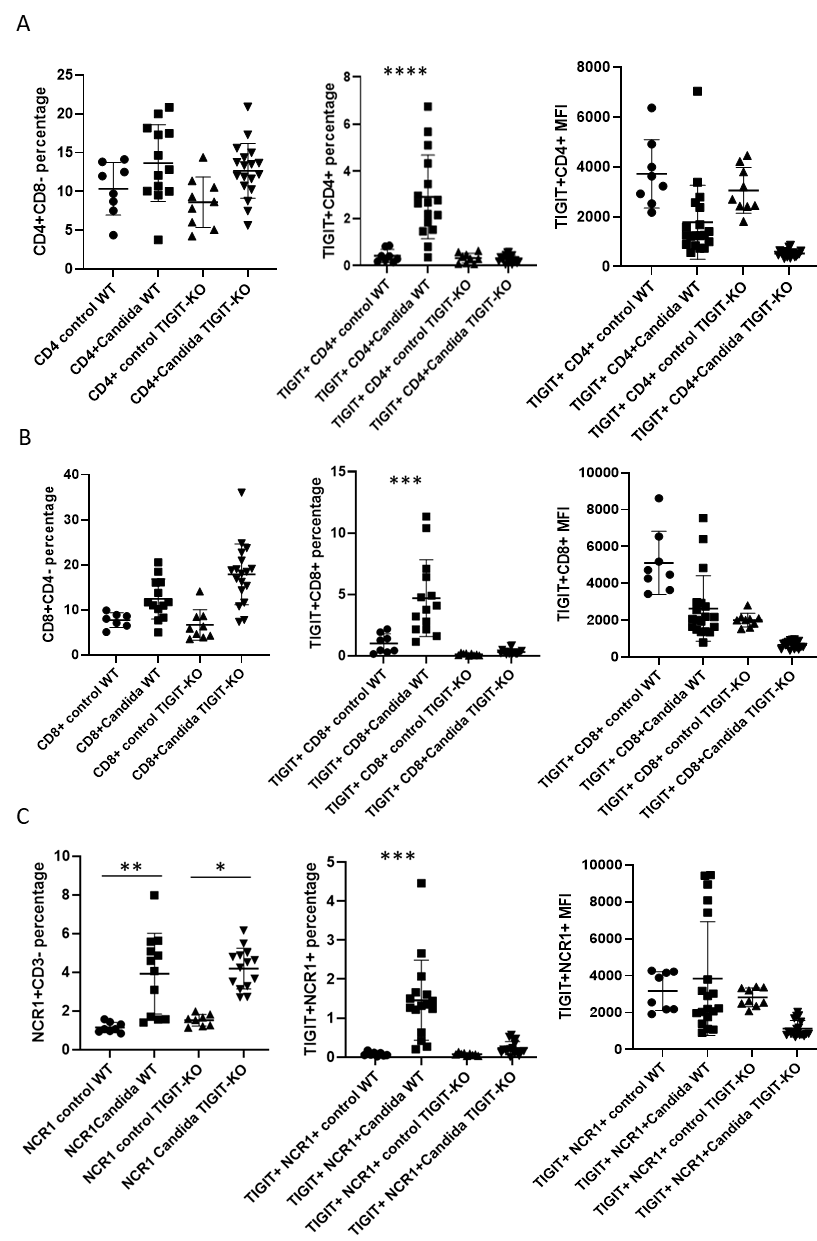


**Fig. S4.** Flow cytometry results analysis of the percentages of CD4, CD8, and NK cells, as well as TIGIT expression, were obtained for kidneys in both healthy and *C. albicans*-infected WT and TIGIT-KO mice. A. Displays the CD4+CD8- percentage in both WT and TIGIT-KO mice (left panel), CD4+TIGIT+ percentage (middle panel) and CD4+TIGIT+ MFI (right panel) before or after *C. albicans* infection. B. Illustrates CD8+CD4- percentage in both WT and TIGIT-KO mice (left panel), CD8+TIGIT+ percentage (middle panel) and CD8+TIGIT+ MFI. And C. Shows NCR-1+CD3- percentage in both WT and TIGIT-KO mice (left panel), CD4+TIGIT+ percentage (middle panel), and CD4+TIGIT+ MFI (right panel). *n* = 7–18 animals examined over 3 independent experiments. *P < 0.05, and **P ≤ 0.01, and *** P ≤ 0.001.


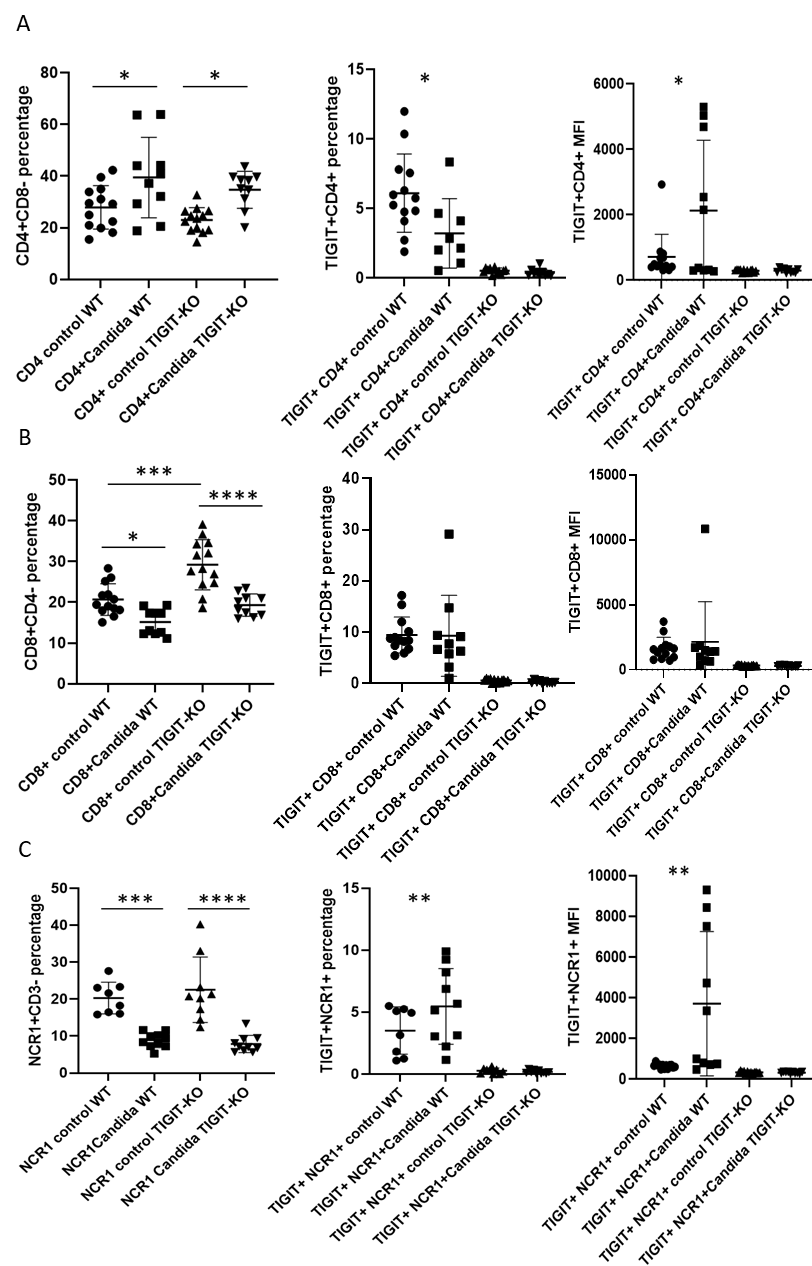


**Fig. S5.** Flow cytometry results analysis of the percentages of CD4, CD8, and NK cells, as well as TIGIT expression, were obtained for the liver in both healthy and *C. albicans*-infected WT and TIGIT-KO mice. A. Displays the CD4+CD8- percentage in both WT and TIGIT-KO mice (left panel), CD4+TIGIT+ percentage (middle panel) and CD4+TIGIT+ MFI (right panel) before or after *C. albicans* infection. B. Illustrates CD8+CD4- percentage in both WT and TIGIT-KO mice (left panel), CD8+TIGIT+ percentage (middle panel) and CD8+TIGIT+ MFI. And C. Shows NCR-1+CD3- percentage in both WT and TIGIT-KO mice (left panel), CD4+TIGIT+ percentage (middle panel) and CD4+TIGIT+ MFI (right panel).    *n* = 8–13 animals examined over 4 independent experiments. *P < 0.05, and **P ≤ 0.01, and *** P ≤ 0.001.
